# Hypervirulent clonal complex (CC) of *Listeria monocytogenes* in fresh produce from urban communities

**DOI:** 10.3389/fmicb.2024.1307610

**Published:** 2024-01-29

**Authors:** Nirosha Ruwani Amarasekara, Amrita Subramanya Swamy, Sumit Kumar Paudel, Wentao Jiang, KaWang Li, Cangliang Shen, Yifan Zhang

**Affiliations:** ^1^Department of Nutrition and Food Science, Wayne State University, Detroit, MI, United States; ^2^Davis College, Division of Animal and Nutritional Sciences, West Virginia University, Morgantown, WV, United States

**Keywords:** *Listeria*, hypervirulence, clonal complex, fresh produce, farmer’s market

## Abstract

**Introduction:**

This study aimed to determine the prevalence and virulome of *Listeria* in fresh produce distributed in urban communities.

**Methods:**

A total of 432 fresh produce samples were collected from farmer’s markets in Michigan and West Virginia, USA, resulting in 109 pooled samples. *Listeria* spp. were isolated and *L. monocytogenes* was subjected to genoserogrouping by PCR and genotyping by pulsed-field gel electrophoresis (PFGE). Multi-locus sequence typing (MLST) and core-genome multi-locus sequence typing (cgMLST) were conducted for clonal identification.

**Results:**

Forty-eight of 109 samples (44.0%) were contaminated with *Listeria* spp. *L. monocytogenes* serotype 1/2a and 4b were recovered from radishes, potatoes, and romaine lettuce. Four clonal complexes (CC) were identified and included hypervirulent CC1 (ST1) and CC4 (ST219) of lineage I as well as CC7 (ST7) and CC11 (ST451) of lineage II. Clones CC4 and CC7 were present in the same romaine lettuce sample. CC1 carried *Listeria* pathogenicity island LIPI-1 and LIPI-3 whereas CC4 contained LIPI-1, LIPI-3, and LIPI-4. CC7 and CC11 had LIPI-1 only.

**Discussion:**

Due to previous implication in outbreaks, *L. monocytogenes* hypervirulent clones in fresh produce pose a public health concern in urban communities.

## 1 Introduction

*Listeria* is widely distributed in the environment, such as soil, manure, and water (Zhu et al., 2017). Due to the high mortality and hospitalization rate of listeriosis (20–30%) (Radoshevich and Cossart, 2018) and the increasing popularity of farmer’s markets in urban communities, it is important to understand the prevalence and public health significance of *Listeria spp.* in fresh produce associated with this sector of agriculture. Recent studies have reported 3.1% to 3.9% of fresh produce contaminated with *Listeria* at farmer’s markets and supermarkets (Li K. et al., 2017; Scheinberg et al., 2017; Roth et al., 2018). While the prevalence of *L. monocytogenes* was generally below 1.9% (Johnston et al., 2006; Korir et al., 2016; Li K. et al., 2017; Scheinberg et al., 2017), none of the research provided molecular information or virulence characteristics of the pathogen from these types of products, which is critical for microbial risk assessment of food contamination.

Of 14 serotypes of *L. monocytogenes*, most are in lineage I (serotypes 1/2b, 3b, 3c, 4b) and lineage II (serotypes 1/2a, 1/2c, and 3a). Serotypes 4b and 1/2a predominate in human and food/environment isolates, respectively (Poimenidou et al., 2018). Lineage III and lineage IV are common in animal sources (Shen et al., 2013) and comprise serotypes 4a, some 4b strains, and 4c. *L. monocytogenes* can be further categorized into clonal complexes (CCs) based on multi-locus sequence typing (MLST). CC1, CC2, CC4, and CC6 of serotype 4b are remarkably more common in human listeriosis than food isolates and considered hypervirulent clones (Maury et al., 2016). Targeting hundreds of core genes of the entire *L. monocytogenes* genome, core genomic MLST (cgMLST) can determine the relatedness of isolates to epidemic clones (EC) and demonstrate the clinical relevance of bacteria (Chen et al., 2016; Li Z. et al., 2017). So far four major ECs (ECI, ECII, ECIII, and ECIV) have been identified in *L. monocytogenes*. ECI, ECII, and ECIV belong to serotype 4b while ECIII belongs to serotype 1/2a (Chen and Knabel, 2007).

*Listeria* pathogenicity island (LIPI) is a key virulence determinant in *Listeria* (Disson et al., 2021). LIPI-1 is listeriolysin O (LLO) island and necessary for intracellular survival and spread of *L. monocytogenes*; LIPI-2 is specific to *L. ivanovii*; LIPI-3 is listeriolysin S island, important for gastrointestinal colonization, and commonly associated with lineage I; LIPI-4 is the most recently identified neurovirulence/placental infection island (Maury et al., 2016) and strongly associated with the hypervirulence of CC4. LIPI-4 has been detected in CC4 as well as emerging clones of human and environmental origin (Lee et al., 2018). Another virulence feature that is crucial for the infectious potential of *Listeria* is the gene clusters responsible for teichoic acid biosynthesis. Teichoic acids are the most abundant glycopolymers in *Listeria* cell wall and play an important role in biofilm formation, phage susceptibility, antimicrobial resistance, and virulence (Sumrall et al., 2020). Serotype-specific glycosylation of teichoic acids mediated by different genes can lead to variation in virulence potential across clonal groups. Of the most common genes reported, *gltA* and *gltB* are specific for serotype 4b (Lei et al., 2001). *gtcA* has been found in serotype 4b and 1/2a (Cheng et al., 2008). *Listeria* internalin A encoded by *inlA* is a key factor mediating bacterial adhesion and invasion of host cells. Full-length *inlA* confers stronger virulence than *Listeria* harboring a premature stop codon (PMSC), the latter of which is 10,000-fold less invasive (Chen et al., 2011). While *inlA* and *inlB* are both important in intestinal and placental barrier invasion, *inlB* also plays a critical role in neuroinvasion and bacterial persistence (Maudet et al., 2022).

Considering the increasing popularity of farmer’s markets in urban communities, it is critical to understand *Listeria* contamination in locally grown fresh produce and their public health significance. Even though several studies reported the prevalence of *Listeria* in fresh produce obtained from farmer’s markets as discussed above, none described the virulence characteristics of bacteria. Therefore, this study aimed to determine the clonal identity and virulome of *L. monocytogenes* in fresh produce obtained from multiple farmer’s markets as related to human infections based on whole-genome sequencing (WGS) and molecular analysis.

## 2 Materials and methods

### 2.1 Sample collection, bacteria isolation, and identification

A total of 432 fresh produce samples were collected from six farmer’s markets (A through F) in Michigan and West Virginia in the summer of 2019. The sites in Michigan were in the metro Detroit area and those in West Virginia in the Morgantown area. The two areas were approximately 400 miles apart. Markets A, C, D, and E mainly sold plant-based products, such as produce, herbs, beans etc. Markets B and F had a diverse collection of vendors that sold a large variety of items, including produce, meats, eggs, spices, etc. Convenient samples were taken from different produce vendors each week for the entire summer. For *Listeria* isolation, 50 grams of each vegetable sample was mixed with 450 mL of buffered listeria enrichment broth (BD, Sparks, MD, USA) and manually homogenized for 2 min. Three to six individual rinses of the same category were pooled and resulted in 109 pooled samples (Salazar et al., 2021; Hitchins et al., 2022). These included 41 leafy greens (romaine lettuce, collards, celery, basil, and kale), 39 root vegetables (radish, turnip, carrot, potato, beet, and onion), and 29 seeded vegetables (cantaloupe, tomato, cucumber, and zucchini).

Bacteria isolation followed the FDA Bacteriological Analytical Manual (BAM) method. One hundred milliliters of vegetable rinse were incubated at 30°C for 4 h for pre-enrichment, followed by the addition of selective agents, acriflavine (10 mg/L) and nalidixic acid (40 mg/L), and further incubated for 48 h at 30°C. The enriched broth was spread on PALCAM *Listeria* agar (Remel Inc., Lenexa, KS, USA) at 24 h and 48 h of incubation. Plates were incubated at 30°C for 24 h before presumptive *Listeria* colonies were streaked onto Rapid L’ mono differentiation agar (Bio-Rad, Hercules, CA, USA). PCR was conducted to identify *Listeria* spp., *L. monocytogenes*, and *L. monocytogenes* serotypes 1/2a, 1/2b, and 4b. *L. monocytogenes* ATCC 51773 was used as positive control. A no template control was included as well. PCR parameters and primer sequences were followed as described by Chen et al. (2017). The Chi-Square test of independence and the *Post-Hoc* tests were performed using SPSS v29.0.1 to compare *Listeria* prevalence in different sites and the significant association at 0.05 significance level.

### 2.2 Pulsed-field gel electrophoresis (PFGE)

Pulsed-field gel electrophoresis was carried out following the standardized protocol for *L. monocytogenes* (Graves and Swaminathan, 2001). Genomic DNA was prepared by mixing 240 μl of standardized cell suspension and 60 μl of 20 mg/ml lysozyme solution (ThermoFisher Scientific, Wilmington, DE, USA) followed by incubation at 37°C for 10 min. Sample plugs were digested with 25 U of *Asc*I (New England Biolabs, Ipswich, MA, USA) at 37°C for 4 h. Plugs were then loaded on 1.2% Megabase agarose gel (Bio-rad) and electrophoresed on a CHEF- DR III apparatus (Bio-rad) using the following parameters: initial switch time, 4 s; final switch time, 40 s; run time, 22 h; angle, 120 ; gradient, 6 V/cm; temperature, 14°C; ramping factor, linear. PFGE patterns were analyzed using Bionumerics (version 6.6; Applied Maths, Austin, TX, USA). Indistinguishable profiles were defined as those demonstrating the same number and sizes of DNA fragments.

### 2.3 Whole genome sequencing (WGS)

Whole genome sequencing was conducted at Omega Bioservices (Norcross, GA, USA). Briefly, DNA was extracted using the E.Z.N.A.^®^ Bacterial DNA Kit (Omega Bio-tek, Norcross, GA, USA). The concentration was measured using the QuantiFluor dsDNA System on a Quantus Fluorometer (Promega, Madison, WI, USA). A Kapa Biosystems HyperPlus kit (Kapa Biosystems, Wilmington, MA, USA) was used for the whole-genome library construction. DNA was fragmented, and ends were repaired, 3’ adenylated, and ligated to adapters. The resulting adapter-ligated libraries were PCR-amplified. After Illumina indexes were added, they were pooled for multiplexed sequencing on an Illumina X10 platform (Illumina, San Diego, CA, USA) using the pair-end 150 bp run format. *de novo* assembly was performed on Ridom SeqSphere + v9.0 (Ridom GmbH, Germany) using SKESA 2.4.0 (Souvorov et al., 2018).

### 2.4 MLST and cgMLST

*In silico* MLST was performed to identify clonal complex (CC) and sequence type (ST) on CLC genomic workbench (Qiagen, Redwood City, CA, USA) using seven housekeeping genes of *L. monocytogenes*, including ABC transporter (*abcZ*), beta-glucosidase (*bglA*), catalase (*cat*), succinyl diaminopimelate dessucinylase (*dapE*), d-amino acid aminotransferase (*dat*), l-lactate dehydrogenase (*ldh*), and histidine kinase (*lhkA*). An allele number was assigned to the sequence of each allele and CC determined based on the definition of Ragon et al. (2008) and the Pasteur MLST database.^1^ WGS data were further subjected to cgMLST cluster identification on Ridom SeqSphere + v9.0 (Ridom GmbH, Germany) (*L. monocytogenes* cgMLST scheme, 1701 loci) (Ruppitsch et al., 2015). The cluster distance threshold for the core genome was 10 allele differences. Epidemic clones of ECI, ECII, ECIII and ECIV (Chen and Knabel, 2007) were downloaded from the NCBI website.

### 2.5 Gene profiling

Virulence genes and antibiotic resistance genes were identified against the BIGSdb-*Lm* database (Moura et al., 2016). The heatmap was generated based on the presence or absence of a virulence gene using Morpheus matrix visualization and analysis software from the Broad Institute.^2^ The WGS data were also screened for stress islands as well as biocide tolerance genes for benzalkonium chloride (*bcrABC, emrCE*) and quaternary ammonium compounds (*qacA*), which are the most common biocides used for *L. monocytogenes*. The coding sequence of *inlA* was examined to ascertain whether the isolates carried a complete sequence or a mutation leading to a PMSC. Nucleotide sequences were aligned with *inlA* of *L. monocytogenes* EGD-e (NCBI: NC_003210.1) as reference.

## 3 Results

### 3.1 Prevalence of *Listeria* and *L. monocytogenes* in fresh produce

All six markets had at least one sample positive for *Listeria* (Table 1). Site A had the highest *Listeria* prevalence (100%) (*p* < 0.05) and site B the lowest (28.6%, *p* < 0.05). *Listeria* spp. were recovered from 48 samples (44.0%), of which 32 were contaminated by *L. welshimeri*, 13 by *L. innocua*, and three by *L. monocytogenes*. All isolates from sites A, D, and E were *L. welshimeri*. Sites B and F had three *Listeria* species identified. *L. monocytogenes* was detected in sites B and F, the two markets that sold both vegetables and animal products such as meat and eggs. The two sites also had the highest number of samples collected, 49 from B and 34 from F, total accounting for more than 76% of 109 pooled samples. *L. monocytogenes* isolates included five each from potato and radish on site B and two from romaine lettuce on site F. All five isolates from radish (43a-e) and one from romaine lettuce (V11c) belonged to serotype 4b and the remaining six from potato (52a-e) and romaine lettuce (V11a) were identified as serotype 1/2a.

**TABLE 1:** Prevalence of *Listeria* spp. in fresh produce from farmer’s markets.

Site	No. of pooled samples	No. of *Listeria* + samples (%)	*Listeria* spp. (%)		
			***L. welshimeri***	***L. innocua***	***L. monocytogenes***
A	6	6 (100)	6 (100)	0 (0)	0 (0.0)
B	49	14 (28.6)	11 (22.5)	1 (2.0)	2 (4.1)
C	6	4 (66.6)	2 (33.3)	2 (33.3)	0 (0.0)
D	4	2 (50.0)	2 (50)	0 (0.0)	0 (0.0)
E	10	3 (33.3)	3 (33.3)	0 (0.0)	0 (0.0)
F	34	19 (55.8)	8 (23.5)	10 (29.4)	1 (2.9)
Total	109	48 (44.0)	32 (29.36)	13 (11.93)	3 (2.75)

### 3.2 Clonal relatedness of *L. monocytogenes*

**FIGURE 1:**
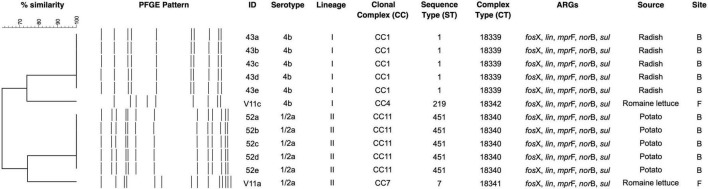
PFGE dendrogram of *L. monocytogenes* from fresh produce. ARG denotes antibiotic resistance gene.

Four unique PFGE patterns were identified in *L. monocytogenes* (Figure 1). Isolates of the same serotype fell into the same cluster. Radish isolates 43a-e of serotype 4b shared the same DNA pattern and were clustered together with V11c from romaine lettuce. Similarly, 52a-e of serotype 1/2a from potato were indistinguishable from each other and clustered together with V11a.

### 3.3 MLST and cgMLST analysis

**FIGURE 2:**
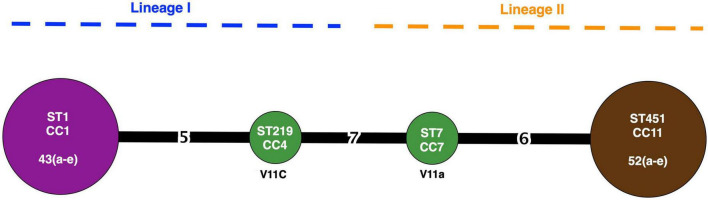
CC, ST, and lineage distribution of *L. monocytogenes* based on MLST. Node size represents number of isolates. Number on the connecting line represents allelic differences. Node color represents source of isolates: purple (radish), green (romaine lettuce), and brown (potato).

**FIGURE 3:**
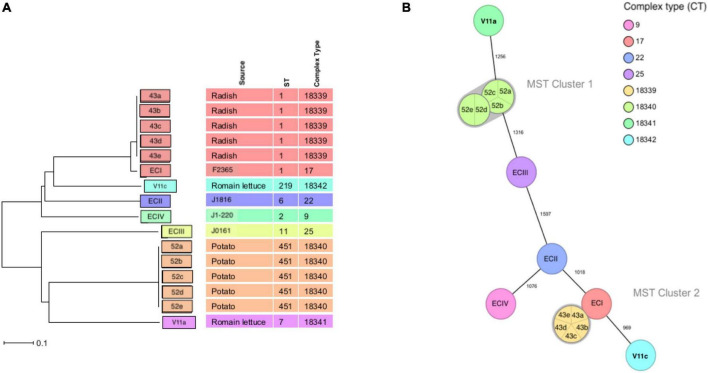
Minimum spanning tree of cgMLST analysis. **(A)** Minimum Spanning Tree (MST) and **(B)** Neighbor Joining Tree (NJT) illustrating the phylogenetic relationship based on allelic profiles of *L. monocytogenes*. MST cluster threshold is 10. Numbers on connecting lines represent the number of allelic differences between two strains.

Twelve isolates that were subjected to WGS had a sequencing coverage of 35 × to 50 × and a quality score above 30 (or base call accuracy 99.9%). Gene sequence data were used for downstream MLST and cgMLST analysis. *In silico* MLST revealed four CCs and STs (Figure 2). A 43a-e and V11c of serotype 4b (lineage I) belonged to CC1 (ST1) and CC4 (ST219), respectively, whereas V11a and 52a-e of serotype 1/2a (lineage II) fell under CC7 (ST7) and CC11 (ST451), respectively. The two isolates from romaine lettuce were designated as different CCs (CC4 and CC7). The hypervirulent CC1 and CC4 differed by five alleles out of seven target genes. The four CCs identified by MLST corresponded to four complex types (CT) by cgMLST (Figures 1, 3). Isolates 43a-e and 52a-e belonged to CT18339 and CT18340, respectively. Two isolates from romaine lettuce were split into CT18341 (isolate V11a) and CT18342 (isolate V11c) and belonged to two distinct clusters (Figure 3). V11a and 52a-e were in cluster 1. Isolates 52a-e were further divided into two groups by one allelic difference. V11c and 43a-e were in cluster 2, along with epidemic clones ECI, ECII and ECIV. Belonging to different CTs, 43a-e and ECI were closely related bearing only 68 allelic differences out of 1701 target loci. V11c and ECI differed by 969 alleles.

### 3.4 Virulome and antibiotic resistance genes

**FIGURE 4:**
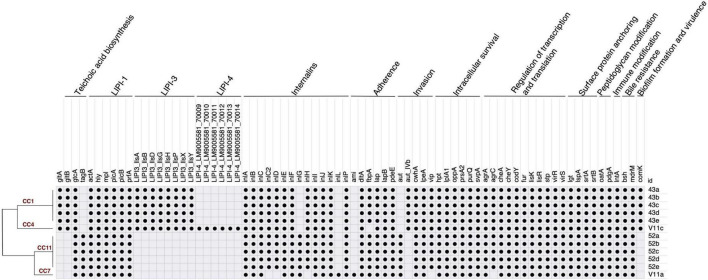
Virulence gene profile of *L. monocytogenes* from fresh produce. 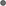 denotes the presence of virulence gene.

The number of virulence genes ranged from 53 to 70 per isolate (Figure 4). Identical gene profile existed in isolates 43a-e, and so did in 52a-e. LIPI-1 was detected in all isolates. The major difference between lineage I and II was in LIPI-3, LIPI-4, and genes for teichoic acid biosynthesis. LIPI-3 was detected in serotype 4b CC1 and CC4. LIPI-4 was carried by CC4 only. Of the four genes for teichoic acid biosynthesis, *gtcA* was identified in both serotypes. *gltA* and *gltB* were found in serotype 4b, whereas *tagB* was contained by serotype 1/2a. Other virulence factors included internalin genes and those involved in various steps of infection such as adherence, invasion, intracellular survival, regulation of transcription and translation, surface protein anchoring, peptidoglycan modification, immune modification, bile resistance, and biofilm formation. All isolates had internalin genes *inlABCEFIJKP*. No PMSC was detected in *inlA*. *inlG* was found in serotype 1/2a but not 4b. CC7 (isolate V11a) was the only one carrying *inlL* but lacking *inlC2DH*. This isolate also had *ami* for adherence that was missing from all other isolates. Another notable difference was the invasion gene *aut* that was found in lineage II whereas its variant (*aut_IVb*) was carried by lineage I. Serotype 4b isolates also contained *comK*, a gene presumably for biofilm formation and persistence in food processing facilities (Hurley et al., 2019). V11a was found to harbor stress survival islet-1 (SSI-1), while none of the other isolates exhibited any stress islands. Biocide tolerance genes were not detected in any isolates. All isolates shared the same antibiotic resistance gene profile that included *fos*X (fosfomycin), *lin* (lincomycin), *mpr*F (defensin), *nor*B (fluoroquinolone), and *sul* (sulfonamide).

## 4 Discussion

*Listeria* are commonly present in the soil of various agricultural landscapes, thus freshly harvested produce is at a high risk of contamination (Chapin et al., 2014). Because vegetables sold at farmer’s markets have gone through limited processing and packaging procedures, any microbial contamination at farm level would end up in the final product. The recovery of CC4 (ST219) in fresh produce in this study is of public health concern. Considering the hypervirulence potential of this clonal group and the ready-to-eat nature of fresh produce, it is important to monitor the prevalence of hypervirulent clones of *L. monocytogenes* in urban communities and understand their clinical relevance.

*Listeria* prevalence in fresh produce varied across previous studies. Research on farmer’s markets in West Virginia and Kentucky (Li K. et al., 2017) as well as Pennsylvania (Scheinberg et al., 2017) both revealed the prevalence of slightly below 4%. An earlier study conducted in the Washington D.C. area demonstrated 19.7% of fresh produce contaminated with *Listeria* (Thunberg et al., 2002). In comparison, our study found *Listeria* at 44.0% (48 of 109 samples). This high prevalence can be due to the isolation procedure as most studies (Li K. et al., 2017; Scheinberg et al., 2017) used individual samples for bacteria isolation whereas others (Thunberg et al., 2002) and our study used pooled samples. The overall 2.8% of *L. monocytogenes* prevalence in this study is in line with previous research where the contamination level in fresh produce ranged from 0.66% to 4.7% (Thunberg et al., 2002; Li K. et al., 2017; Scheinberg et al., 2017; Roth et al., 2018). The market variation on *L. monocytogenes* prevalence (*p* > 0.05) can be explained by the predominance of samples in markets B and F, although cross contamination from meat cannot be excluded at large-scale markets with a diversity of vendors. In fact, multiple commodities sold at farmer’s markets, including meat, have been found contaminated with *L. monocytogenes* (Scheinberg et al., 2017; Kim et al., 2021). *L. welshimeri* and *L. innocua* appeared to be the most common *Listeria* species in fresh produce and have been reported previously (Thunberg et al., 2002).

The detection of *L. monocytogenes* 1/2a and 4b in fresh produce indicates a food safety concern given that 1/2a, 1/2b, and 4b are the top three serotypes responsible for human listeriosis (Liu, 2006). Isolates from radish and potato can be considered identical clones due to demonstrating indistinguishable profiles. The one-allelic difference between 52a-c and 52d-e from potato based on cgMLST should be negligible as the isolates shared otherwise identical profiles, including virulence genes and antibiotic resistance genes. Two genetically distinct clones in romaine lettuce may suggest different sources of contamination and the variation in virulence potential, although it is also possible that the two isolates were from two individual samples before being pooled for bacteria isolation. All four clonal groups identified in this study have been implicated in foodborne outbreaks worldwide (Chen et al., 2016). CC1 was formerly designated ECI because of its previous outbreak association. Their close connection is reflected by isolates 43a-e of this study differing from ECI by only 68 alleles. CC4 seemed to have mainly caused outbreaks in Europe (Dorozynski, 2000; Tasara et al., 2015; Chen et al., 2016), yet its recovery from local fresh produce should be highlighted because of its strong association with the central nervous system and maternal-neonatal listeriosis (Maury et al., 2016). Future surveillance at a larger scale would benefit our understanding of the extent of hypervirulent clones in urban communities. Moreover, while the hypervirulence potential itself could be a factor for the overrepresentation of CC4 in human isolates, non-food exposure should be considered as well because CC4 has been documented in various environmental sources such as animals and surface water (Lee et al., 2018; Raschle et al., 2021). CC7 and CC11 have caused multiple US outbreaks (Olsen et al., 2005; Centers for Disease Control and Prevention, 2011; Jackson et al., 2011). CC11 in association with maternal-neonatal listeriosis has been suggested from outbreaks involving pregnant, newborn, and elderly patients from consuming contaminated deli meat (Olsen et al., 2005) and Mexican-style cheese (Jackson et al., 2011). Taken together, fresh produce distributed in urban communities can be potential sources of *Listeria* clones with serious public health implication and requires close monitoring.

At the gene level, LIPI-1 is present in all *L. monocytogenes* and considered core virulence genes (Disson et al., 2021). LIPI-3 and LIPI-4 are critical for lineage I strains. LIPI-3 was carried by CC1 and CC4 of this study. It comprised genes encoding hemolysin listeriolysin S (*llsABDGHPXY*) and important for gastrointestinal colonization. Strongly associated with the hypervirulence of CC4, LIPI-4 is the most recently discovered pathogenicity island that encodes cellobiose-type phosphotransferase systems (PTS) and enhances neural and placental infection. The distribution of genes involved in teichoic acid biosynthesis is consistent with *Listeria* in Swiss surface water (Raschle et al., 2021) where CC1 and CC4 contained serotype 4b-specific *gltA* and *gltB* but lacked *tagB*, a gene most characterized in *Bacillus subtilis* (Brown et al., 2013). Because *tagB* was one of the genes unique to strong biofilm producers of *L. monocytogenes* (Park et al., 2022), the presence in serotype 1/2a in this study indicates the high potential of biofilm formation by these isolates. Despite that PMSC is commonly found in *Listeria* from food and food production environment (Nightingale et al., 2008; Li Z. et al., 2017), none of the isolates in this study carried PMSC, suggesting a full virulence potential.

The five antibiotic resistance genes detected in this study (*fosX, lin, mprF, norB, sul*) have been reported from various sources worldwide, including ready-to-eat meat (Mafuna et al., 2021; Centorotola et al., 2023), food processing environment (Hurley et al., 2019; Wieczorek et al., 2020), surface water (Raschle et al., 2021), and wildlife (Brown et al., 2023). This is not surprising as *Listeria* are intrinsically resistant to fosfomycin, many modern cephalosporins, oxacillin, sulfonamide, and nalidixic acid (Troxler et al., 2000; Mota et al., 2020; Brown et al., 2023). The genes detected in this study were either known (*fosX*) or strongly suggested (*lin, norB, sul*) to be intrinsic resistance genes. Because the isolates in this study were recovered from geographically and temporally separate occasions, it remains to be tested as to their antibiotic susceptibility phenotypes as genotypes and phenotypes do not always coincide. Although antibiotic resistance in general is not a serious issue in *Listeria*, multidrug-resistant *Listeria* do occur in food (Kayode and Okoh, 2022a,b). Thus, it is important to monitor the prevalence of antibiotic resistance genes, especially in hypervirulence clonal groups.

In conclusion, *Listeria* species are prevalent in vegetables distributed in urban communities. The presence of *L. monocytogenes* clones carrying a range of virulence genes indicates a potential public health concern. It is critical for urban food producers and distributors to control microbial transmission from the environment and other sources at urban gardens and farmer’s markets. Given the remarkable clinical significance of the hypervirulent clones and their recovery in the environment and animals, future research should focus on understanding the source of contamination, including wildlife and animal fertilizers, as well as the contribution of food, animal, and environmental exposure to listeriosis.

## Data availability statement

The datasets presented in this study can be found in online repositories. The names of the repository/repositories and accession number(s) can be found in the article/supplementary material. The *L. monocytogenes* WGS data presented in the study are deposited in the NCBI repository, BioProject PRJNA1006699.

## Author contributions

NA: Data curation, Formal analysis, Investigation, Methodology, Validation, Writing – original draft. AS: Investigation, Writing – review and editing. SP: Investigation, Writing – review and editing. WJ: Investigation, Writing – review and editing. KL: Investigation, Writing – review and editing. CS: Conceptualization, Resources, Supervision, Validation, Writing – review and editing. YZ: Conceptualization, Funding acquisition, Project administration, Resources, Supervision, Validation, Writing – review and editing.

## Footnotes

1
https://bigsdb.pasteur.fr/listeria/

2
https://software.broadinstitute.org/morpheus/
